# The efficiency of insulin production and its content in insulin-expressing model β-cells correlate with their Zn^2+^ levels

**DOI:** 10.1098/rsob.200137

**Published:** 2020-10-21

**Authors:** Petra Dzianová, Seiya Asai, Martina Chrudinová, Lucie Kosinová, Pavlo Potalitsyn, Pavel Šácha, Romana Hadravová, Irena Selicharová, Jan Kříž, Johan P. Turkenburg, Andrzej Marek Brzozowski, Jiří Jiráček, Lenka Žáková

**Affiliations:** 1 Institute of Organic Chemistry and Biochemistry, Czech Academy of Sciences, Flemingovo nám. 2, 116 10 Prague 6, Czech Republic; 2 Department of Biochemistry, Faculty of Science, Charles University, 12840 Prague 2, Czech Republic; 3 Laboratory of Pancreatic Islets, Institute for Clinical and Experimental Medicine, Videnska 1958/9, 140 21 Prague, Czech Republic; 4 York Structural Biology Laboratory, Department of Chemistry, University of York, Heslington, York YO10 5DD, United Kingdom

**Keywords:** β-cells, insulin, pancreatic islets, proinsulin, zinc ions, znt8

## Abstract

Insulin is produced and stored inside the pancreatic β-cell secretory granules, where it is assumed to form Zn^2+^-stabilized oligomers. However, the actual storage forms of this hormone and the impact of zinc ions on insulin production *in vivo* are not known. Our initial X-ray fluorescence experiment on granules from native Langerhans islets and insulinoma-derived INS-1E cells revealed a considerable difference in the zinc content. This led our further investigation to evaluate the impact of the intra-granular Zn^2+^ levels on the production and storage of insulin in different model β-cells. Here, we systematically compared zinc and insulin contents in the permanent INS-1E and BRIN-BD11 β-cells and in the native rat pancreatic islets by flow cytometry, confocal microscopy, immunoblotting, specific messenger RNA (mRNA) and total insulin analysis. These studies revealed an impaired insulin production in the permanent β-cell lines with the diminished intracellular zinc content. The drop in insulin and Zn^2+^ levels was paralleled by a lower expression of ZnT8 zinc transporter mRNA and hampered proinsulin processing/folding in both permanent cell lines. To summarize, we showed that the disruption of zinc homeostasis in the model β-cells correlated with their impaired insulin and ZnT8 production. This indicates a need for in-depth fundamental research about the role of zinc in insulin production and storage.

## 1. Introduction

Insulin is one of the key hormones responsible for carbohydrate homeostasis and metabolic response. Insulin is synthesized in the endoplasmic reticulum (ER) as a 110 amino acid preproinsulin. The cleavage of its signal sequence yields 81 amino acid proinsulin that is transported into the Golgi apparatus, in which the immature—proinsulin-containing—insulin secretory granules (ISGs) are formed that dominate the content of the β-cell [1]. For example, the rodent β-cell contains approximately 10 000 ISGs of 300–500 nm diameter (approx. 10–20% of its total cell volume [2]), with each granule storing about 200 000 insulin molecules [3]. It is envisaged that proinsulin firstly forms there, soluble Zn^2+^-stabilized hexamers, which are finally processed into a fully functional, mature 51 amino acid two-chain insulin molecule that is still held in the Zn^2+^-maintained hexamer [4–7]. It is thought that the proinsulin → insulin hexamer transition lowers the solubility of the resulting insulin oligomers, which subsequently form some crystalline material [8].

The *in vitro* studies showed that different types of insulin hexamers have *in vitro* distinctive thermodynamic stabilities [9,10], hence the prevalence of a specific type of insulin oligomer in the ISGs could have an impact on the insulin pancreas → bloodstream secretion process, i.e. the bioavailability of this hormone. However, despite the abundance of *in vitro* studies of insulin, there is still a lack of direct, experimental *in vivo* evidence for the type of storage form of insulin, e.g. of particular insulin crystals in the β-cells, and the indication of a specific, oligomeric form of the hormone.

This long-standing uncertainty—almost 100 years—about the *in vivo* structural (presumably crystalline) form of the ISG stored insulin, and our long-term interest in the insulin structure–function relationship [11–15] prompted our trials to address this challenge, and to elucidate the quaternary structure of insulin in live ISGs. These trials were encouraged by the development of cutting-edge high-brilliance radiation synchrotron sources and detectors. We initiated this research by the X-ray fluorescence (XRF) analysis of the isolated ISGs from rat pancreas and rat-origin permanent INS-1E β-cells. The selection of the material was also dictated by the need for the most feasible and ethical source of ISGs (INS-1E β-cells) for these very material-demanding studies. Surprisingly, the XRF scans revealed a remarkable difference in Zn^2+^ content between INS-1E and native rat pancreatic islets' ISGs. Although the non-standard insulin production in the permanent insulinoma-derived β-cell lines is well known, the actual lack of Zn^2+^ in their storage granules was unexpected.

These preliminary findings highlighted the need for a much more in-depth focus on Zn-ISG content in the context of the insulin issue, and hence prompted the main research aims addressed in this report: (i) detailed characterization of Zn^2+^ ions’ content in model β-cells, and (ii) elucidation of the role of Zn^2+^ in β-cell insulin production. We performed an in-depth, comparative characterization of different β-cell models: rat-derived permanent INS-1E and BRIN-BD11 cell lines and rat pancreatic islets as a source of native β-cells. Again, the feasibility of the ISG yield and the widespread use of these cell lines were the main factors in their selection. The results obtained in this study revealed a striking correlation between levels of intracellular Zn^2+^ in the studied β-cells and their ability for effective folding and production of insulin, also evoking questions about the causative links between impaired, pathological insulin production and zinc deficiency.

## 2. Results

### 2.1. Isolation of insulin secretory granules

The purpose of the isolation was to obtain the human-homologous, native-like insulin-containing material that was used for the initial analysis of the zinc content by XRF (see below).

**Figure 1.**
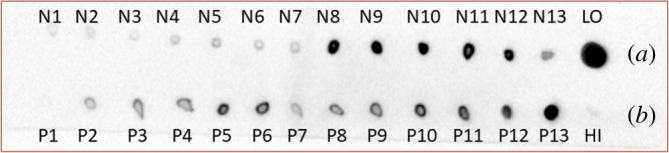
Dot-blot analysis of fractions obtained by the ultracentrifugation of insulin secretory granules from INS-1E cells. Insulin was quantified in each of the 13 fractions obtained by Nycodenz (*a*) or Percoll (*b*) gradients. Fractions N8-N10 and P13 were collected. LO–pancreatic islets, HI–human insulin.

**Figure 2.**
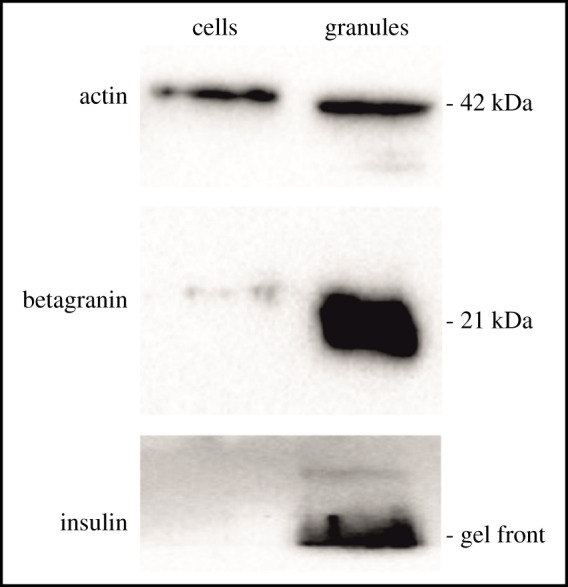
Western blot analysis of INS-1E cells granule fraction preparation. INS-1E cell lysate (2 µg) and isolated granule fraction (2 µg) were separated on 12% gel and tested with anti-actin, anti-chromogranin A and anti-insulin antibodies. Two blots developed from the identical sample are shown but with different exposition of related signal intensity (longer for insulin and shorter for actin/betagranin).

The ISGs were isolated by a discontinuous Nycodenz and the subsequent Percoll gradients, where the ISGs were localized on dot-blots by the insulin (L6B10) mouse mAb (no. 8138). Both gradients yielded 13 fractions (figure 1), with fractions N8 and N9 of the Nycodenz gradient showing the largest amount of insulin (figure 1*a*). Hence they were separated further by the Percoll gradient. The highest level of insulin was observed in fractions P11-P13 (figure 1*b*), which were considered as purified ISGs and used for further experiments. The identity of the ISGs was verified on Western blots using both anti-insulin and anti-chromatogranin A antibodies (figure 2), as 21 kDa betagranin (the N-terminal fragment of chromatogranin A) is one of the characteristic components of ISGs [16].

### 2.2. Transmission electron microscopy

**Figure 3.**
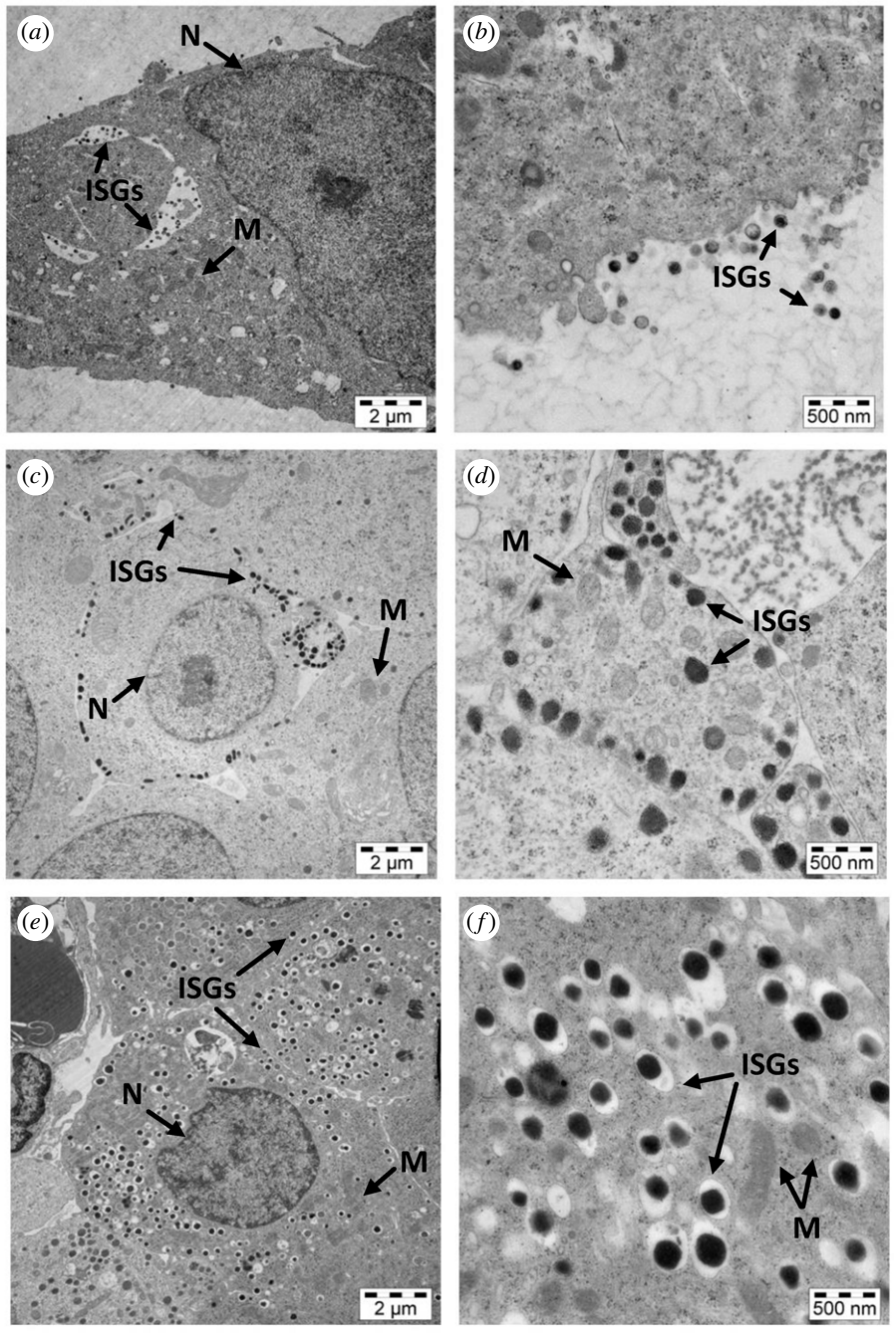
Transmission electron microscopy images of BRIN-BD11 cells (*a,b*), INS-1E cells (*c,d*), and rat pancreatic islets (*e,f*). (*a*,*c* and *e*) Show cells magnified 10 000 times, and images in (*b*,*d* and *f*) were taken with 40 000 × magnification. Arrows in (*a–f*) indicate ISGs commented in the main text; N, nucleus; and M, mitochondria.

Rat pancreatic islets and both permanent INS-1E and BRIN-BD11 cell lines were examined by transmission electron microscopy (TEM). The INS-1E and BRIN-BD11 cell lines displayed a highly reduced number of ISGs (marked by arrows in figure 3*a–d*) in comparison with the rat islet cells (figure 3*e,f*). They also lacked a clear visible halo, which is characteristic of mature ISGs. The absence of a dense core formed by processed insulin surrounded by a lucent and well-defined halo could be a marker of the presence of proinsulin, which is typically localized in the peri-nuclear region of the ISGs [17]. The permanent cell ISGs are also substantially smaller than rat islets ISGs. The size of rat ISGs was between 250 and 300 nm, while the diameter of INS-1E cells' granules was only between 150 and 200 nm, with the size of some BRIN-BD11 ISGs even below 100 nm.

Rat pancreatic islets displayed a large number of β-cells with a dense core, mature ISGs and a translucent halo (marked by arrows in figure 3*e*). Some immature ISGs, slightly larger in size than mature ISGs, and with a low, or intermediate, density (marked by arrows in figure 3*f*) were also observed there. The scattered lamellar ER, Golgi apparatus and numerous mitochondria were also well visible in the rat islet β-cells (figure 3).

### 2.3. Analysis of insulin secretory granules from INS-1E cells by X-ray fluorescence

**Figure 4.**
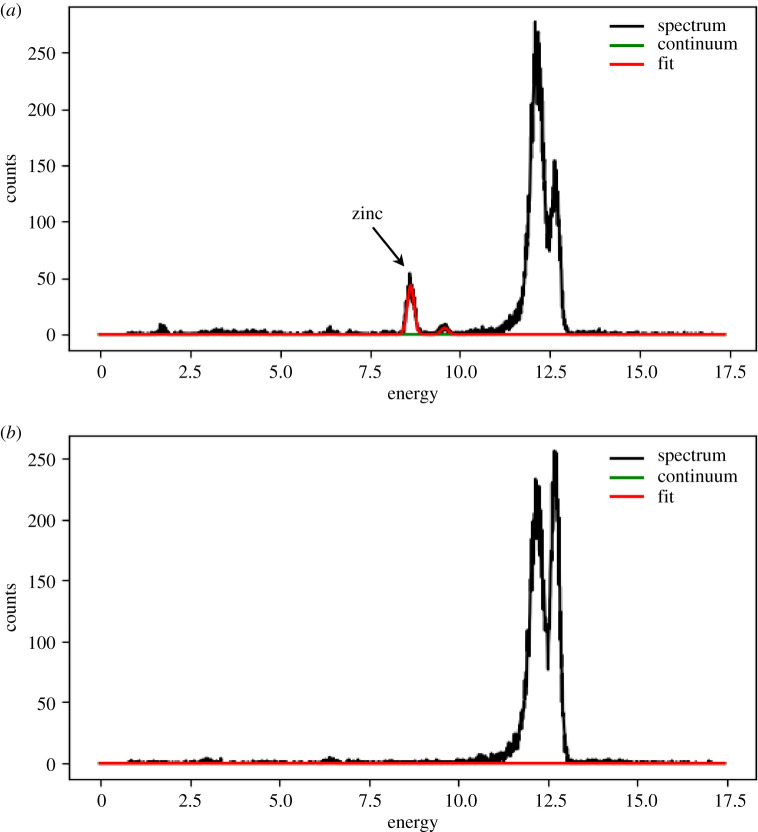
XRF spectra of the ISGs isolated from rat islets (*a*) and INS-1E cells (*b*). In (*a*), the signal of zinc is marked by an arrow. The large double peaks on the right, direct beam; Zn peak, at 8 kV. In red, the fit obtained using PyMCA [18].

The rat and INS-1E β-cells ISGs were also examined by XRF. The XRF scan of the ISGs from the rat β-cells showed the presence of zinc (marked by arrows in figure 4*a*), whereas the XRF scan of the ISG sample from the INS-1E cells did not indicate any presence of these ions (figure 4*b*).

### 2.4. Determination of zinc content in INS-1E cells using atomic absorption spectroscopy (inductively coupled plasma-optical emission spectroscopy)

**Figure 5.**
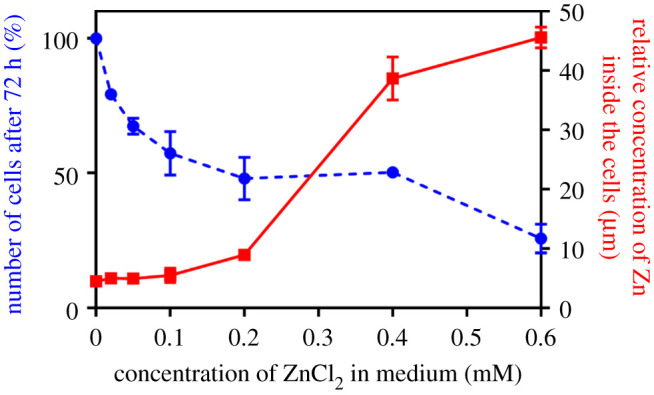
Effects of ZnCl_2_ supplementation on growth of INS-1E cells (number of cells in blue) and on intracellular zinc concentration (in red).

The Zn^2+^ content in the INS-1E cells was investigated by inductively coupled plasma-optical emission spectroscopy (ICP-OES), where the impact of media-zinc supplementation on their intracellular Zn^2+^ concentration and their growth was evaluated. The 72 h exposure of the cells to ZnCl_2_-supplemented (0.0–0.6 mM) medium gave a sigmoidal Zn^2+^ concentration-dependent intracellular increase of these ions (figure 5). This increase was most significant within the 0.2–0.4 mM concentration of ZnCl_2_ in the medium. However, the increase of Zn^2+^ in the medium also had a negative effect on the growth and proliferation abilities of the INS-1E cells. Their numbers decreased rapidly with increasing zinc concentrations, reaching a plateau within the 0.2–0.4 mM Zn^2+^ range, declining finally to approximately 10% of their initial number at 0.4–0.6 mM Zn^2+^. The concentration of Zn^2+^ above 0.8 mM was lethal to the cells. Subsequently, this increasing Zn^2+^ cell toxicity helped to establish its low—50 µM ZnCl_2_—level of the INS-1E and BRIN-BD cells’ growth media for the subsequent flow cytometry and confocal fluorescence microscopy experiments.

### 2.5. Flow cytometry

Fluorescent cell-permeant FluoZin-3AM high Zn^2+^ affinity probe (*K*_d_ value approx. 15 nM) was used for the visualization of the intracellular Zn^2+^ in flow cytometry experiments in the INS-1E, BRIN-BD11 cells, and in the dispersed native rat pancreatic cells. The effect of the cell-permeable Zn^2+^ chelator TPEN (*K*_d_ approx. 3.8 × 10^−15^ M) [19] on the quenching of FluoZin-3AM was also investigated. The fluorescence intensity of the untreated cells (autofluorescence) was determined first, followed by determination of the fluorescence of the TPEN- and, subsequently, FluoZin-3AM-pretreated cells. Finally, the fluorescence of FluoZin-3AM-only-treated cells was measured as well.

**Figure 6.**
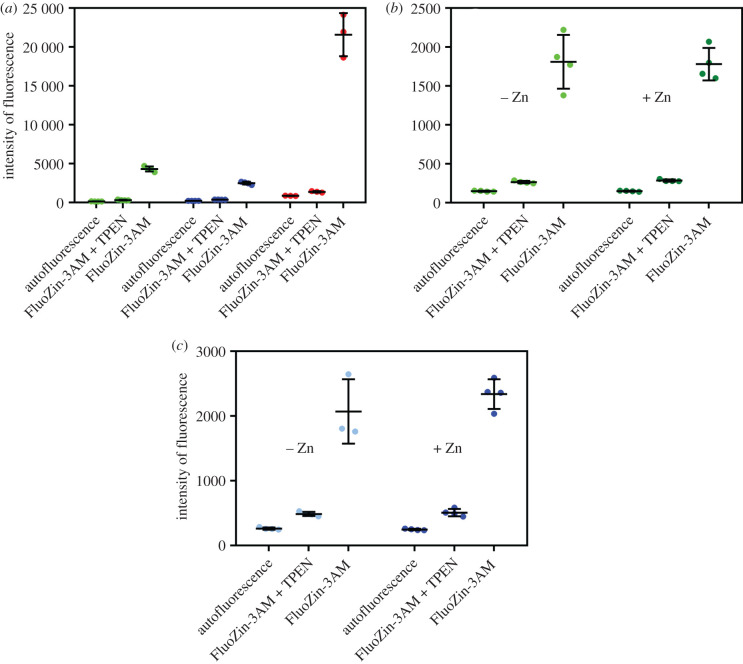
Flow cytometry experiments. (*a*) Intensity of fluorescence of INS-1E cells (green), BRIN-BD11 cells (blue) and rat native pancreatic islet cells (red). Comparison of the intracellular zinc content in INS-1E (*b*) and in BRIN-BD11 cells (*c*) measured as the intensity of fluorescence of cells grown in medium supplemented with (darkly coloured bars) or without (lightly coloured bars) 50 µM ZnCl_2_. The left bar in each light or dark colour set shows autofluorescence in untreated cells, the middle bar, fluorescence of the cells treated with TPEN/FluoZin-3AM and the right bar, fluorescence in the cells treated by FluoZin-3AM only.

All types of cells showed an increase in fluorescence intensity after treatment with FluoZin-3AM, when compared to the basal autofluorescence of the untreated cells, with a substantial reduction in FluoZin-3AM fluorescence in all cell types after pre-treatment with zinc chelator TPEN (table 1). The intensity of FluoZin-3AM fluorescence of rat pancreatic β-cells was approximately 5–8 times higher than the intensity of fluorescence in INS-1E or BRIN-BD11 cells, respectively (table 1, figure 6*a*). To assess the effect of the supplementation of the cells by additional Zn^2+^ ions the above-mentioned fluorescences were also measured in the untreated and 50 µM ZnCl_2_ pretreated INS-1E and BRIN-BD11 cells. Here, this Zn-enrichment of the media had a negligible effect on FluoZin-3AM-related cell fluorescence (figure 6*b,c*).

**Table 1.** Autofluorescence of INS-1E, BRIN-BD11 and rat pancreatic islet cells, and their fluorescence after treatment with FluoZin-3AM/TPEN or with FluoZin-3AM probe only. (The data are the means of four or three independent flow cytometry experiments (±s.d.).)

type of fluorescence signal	relative fluorescence intensity in		
	INS-1E cells	BRIN-BD11 cells	rat pancreatic islet cells
autofluorescence	140 ± 7 (4)	216 ± 8 (4)	867 ± 22 (3)
FluoZin-3AM	4311 ± 320 (4)	2499 ± 174 (4)	21590 ± 2768 (3)
FluoZin-3AM + TPEN	308 ± 51 (4)	371 ± 15 (4)	1370 ± 96 (3)

### 2.6. Confocal fluorescence microscopy

**Figure 7.**
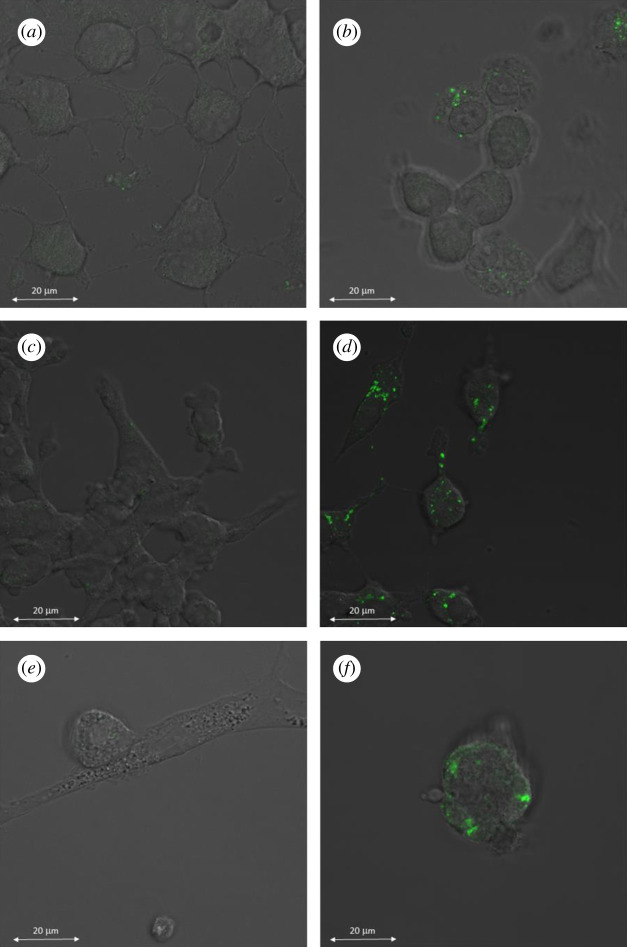
Confocal microscopy images of BRIN-BD11 cells (*a,b*), INS-1E cells (*c,d*), and rat pancreatic islets (*e,f*): pretreated with TPEN (*a,c,e*) or treated with FluoZin3-AM only (*b,d,f*).

Cell-permeant fluorescent probe FluoZin-3AM was also employed to detect the intracellular zinc ions by confocal fluorescence microscopy (CFM), with zinc chelator TPEN used again as a ‘negative’ control. Confocal images of the cells (INS-1E, BRIN-BD11 and dispersed rat pancreatic islet cells) were taken after staining with FluoZin-3AM only (figure 7*b,d,f*), or in the co-presence of TPEN and FluoZin-3AM (figure 7*a,c,e*).

The cells revealed significant differences by CFM in their intracellular zinc content, with the BRIN-BD11 cells showing the lowest, and the islet cells the highest fluorescence. The use of TPEN reversed the FluoZin-3AM-dependent fluorescence in all types of cells to its almost basal levels.

### 2.7. Tricine-sodium dodecyl sulphate--polyacrylamide gel electrophoresis and Western blot analysis of insulin in cell samples

**Figure 8.**
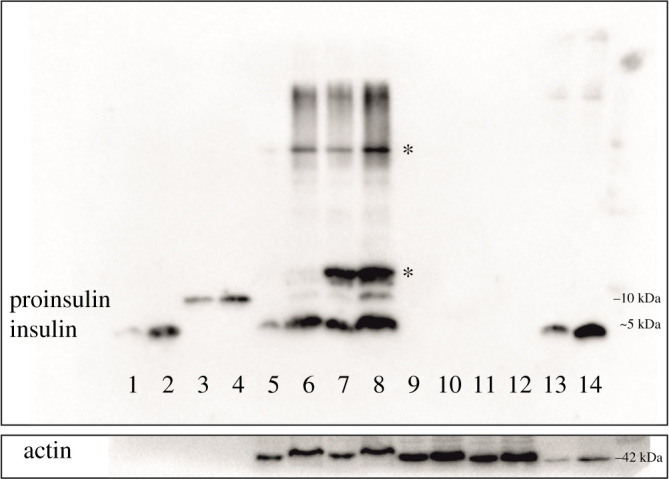
Western blot analysis of proinsulin and insulin content in rat pancreatic islets, INS-1E and BRIN-BD11 cells on the non-reducing tricine-SDS-PAGE separation. Line 1, human insulin (7 ng); line 2, human insulin (13 ng); line 3, bovine proinsulin (7 ng); line 4, bovine proinsulin (13 ng); line 5, INS-1E cells homogenate (1.5 µg); line 6, INS-1E cells homogenate (3 µg); line 7, zinc-supplemented INS-1E cells homogenate (1.5 µg); line 8, zinc-supplemented INS-1E cells homogenate (3 µg); line 9, BRIN-BD11 cells homogenate (1.5 µg); line 10, BRIN-BD11 cells homogenate (3 µg); line 11, zinc-supplemented BRIN-BD11 cells homogenate (1.5 µg); line 12, zinc-supplemented BRIN-BD11 cells homogenate (3 µg); line 13, rat pancreatic islets homogenate (0.3 µg); line 14, rat pancreatic islets homogenate (0.6 µg). Asterisks mark unidentified insulin-immunopositive bands. Control actin content in the cell lysates is shown at the base of the image. Variable amounts of the proteins were applied for a balanced visualization of the insulin-positive bands.

To align the fluorescence-based assessment of Zn^2+^ presence in the ISGs to the context of their insulin content, the appearance of this hormone in the BRIN-BD11, INS-1E β-cell lines and in the rat pancreatic islets was analysed by Western blotting, after separation of the proteins by non-reducing tricine-sodium dodecyl sulphate-polyacrylamide gel electrophoresis (SDS-PAGE). The insulin content was tested in both 50 µM ZnCl_2_-treated and untreated BRIN-BD11 and INS-1E cells. As expected, insulin was observed in the rat Langerhans islets (figure 7, lines 13 and 14) and in homogenates of the INS-1E cells (figure 7, lines 5 and 6). However, the ZnCl_2_-treated INS-1E cells (figure 8, lines 7 and 8) also showed—besides insulin—the weaker presence of proinsulin, and two higher molecular weight protein bands (marked by asterisks in figure 8) with the intensities comparable to the insulin band. By contrast, the BRIN-BD11 cells did not show any detectable insulin-positive bands (figure 8, lines 9–12).

Besides insulin and proinsulin, the INS-1E cells also contain other unidentified proteins with higher molecular weights (figure 8, proteins marked by asterisks). Despite repeated attempts at N-terminal amino acid sequencing, tryptic digestion/mass spectrometry (MS) analysis or immunoprecipitation, these proteins could not be unequivocally identified. However, the MS data of the proteolytic digests of the lower (10 kDa) band showed only the presence of insulin fragments, and no other proteins (data not shown).

### 2.8. Expression of insulin messenger RNA in cell samples

**Figure 9.**
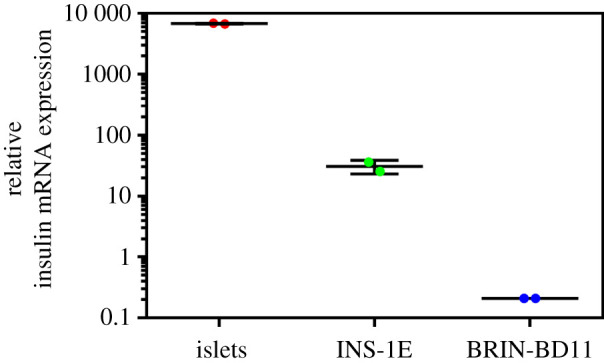
RT-PCR analysis of rat insulin mRNA expression in rat pancreatic islets, INS-1E and BRIN-BD11 cells. The insulin mRNA expression levels (on a logarithmic scale) are related to glyceraldehyde-3-phosphate dehydrogenase mRNA expression.

To achieve an alternative parameter for the assessment of insulin abundance in the studied cells (and the isolated rat pancreatic islets), their insulin messenger RNA (mRNA) expression levels were determined and compared (figure 9). These indicated remarkable differences between a very high expression of insulin mRNA in native rat islets, and approximately 70- and, especially approximately 30 000-fold lower insulin expression in INS-1E or BRIN-BD11 cells, respectively.

### 2.9. Analysis of the total insulin content in permanent cells and Langerhans islets

**Figure 10.**
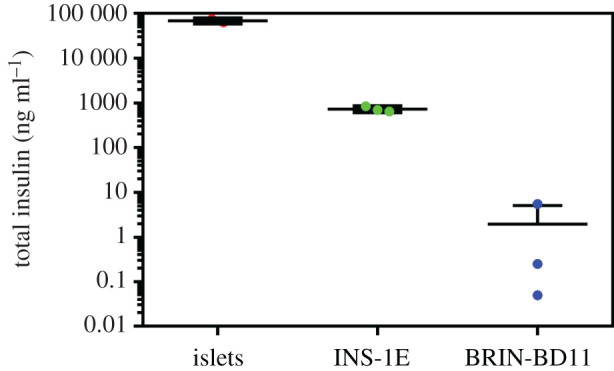
Analysis of the total insulin content in rat pancreatic islets, INS-1E and BRIN-BD11 cells. The total amount of insulin (ng ml^−1^) is shown in a logarithmic scale.

To complete the estimation of insulin in the studied cells and rat islets, they were homogenized after measurements of insulin's mRNA, and their total insulin contents were determined by radioimmunoassay (RIA). The logarithmic scale summary of the total insulin analysis (figure 10) illustrates a remarkable difference between the very high level of insulin in native rat islets and the *ca*. 100- and, especially, approximately 35 000-fold lower level of total insulin in INS-1E or BRIN-BD11 cells, respectively.

### 2.10. Expression of ZnT8 zinc transporter messenger RNA in cell samples

**Figure 11.**
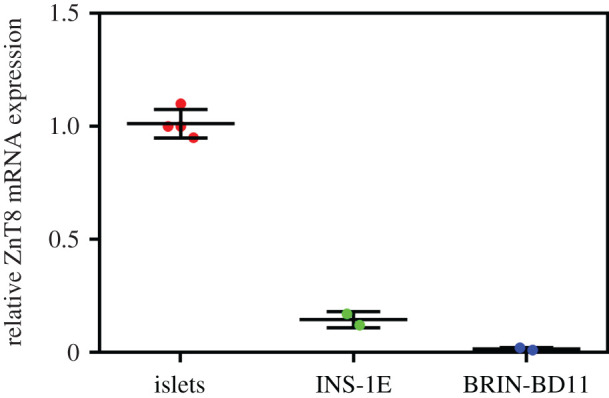
RT-PCR analysis of rat ZnT8 mRNA expression in rat pancreatic islets, INS-1E and BRIN-BD11 cells. The ZnT8 mRNA expression levels are related to glyceraldehyde-3-phosphate dehydrogenase mRNA expression.

Finally, the relative expression levels of the zinc transporter ZnT8 mRNA in isolated rat pancreatic islets, BRIN-BD11 and INS-1E cells were determined to elucidate whether the Zn^2+^ transport is disrupted in these cell lines. This revealed that the expression of ZnT8 in the INS-1E and BRIN-BD11 cells is markedly decreased in comparison with the rat islets (figure 11).

## 3. Discussion

Owing to its central location at the crossroads of metabolism, growth and lifespan regulation, human insulin has been the subject of intense research since the discovery of this hormone in 1921. Despite several hundreds of *in vitro* structures of this hormone, its *in vivo* storage form—in ISGs—has, to date, eluded any characterization. Solid-state and nuclear magnetic resonance studies led to the general assumptions that insulin is stored in the ISGs in some form of zinc-stabilized, crystalline or semi-crystalline hexamers [3,20,21]. Such suppositions were corroborated by reports about high concentrations of both zinc and insulin within the ISGs [5,22], which should favour the formation of insulin hexamer crystals observed *in vitro* in the presence of Zn^2+^ ions. However, there is still a lack of direct *in vivo* experimental evidence for insulin crystals in the β-cells and of the indication of an oligomeric (if any) type of this hormone in ISGs.

We endeavoured to resolve this ambiguity by structural, high-brilliance X-ray studies of live ISGs. However, the preliminary step of this research—XRF of ISGs isolated from rat INS-1E cells and rat islets β-cells—showed a lack of Zn^2+^ ions in the permanent cells, in contrast with a clear Zn-XRF signal from the whole, native islets (figure 4). This unexpected phenomenon revealed the need for an in-depth, systematic biochemical analysis of the cellular material, prior to its further structural studies. Therefore, here we firstly aimed at an analysis of the Zn content in the permanent INS-1E and BRIN-BD11 cells that could be ethical sources of ISGs for material-demanding X-ray experiments and compare it with native rat islets. Second, we attempted an analysis of insulin production in these *in vivo* systems, to learn about a potential relationship between the ISGs Zn^2+^ content on the levels and folding of insulin in these granules.

The permanent β-cell lines derived from insulinomas for the study of β-cell functions were already the subject of several studies [23–28]. Although they showed some experimental limitations of these cells e.g. non-standard insulin production, our initial findings of the potential zinc deficiency in these cells made them very good targets of comparative studies of the presence of intracellular (ISGs) Zn^2+^ in the context of the morphology of insulin production and content. We also employed the same methodologies on the rat pancreatic islets as a source of reference, native β-cells.

Electron transmission micrographs revealed a substantial reduction in the number and size of ISGs in both insulinoma cell lines, especially in BRIN-BD11 cells, in comparison with rat islets (shown by arrows in figure 3). This is in agreement with the already reported low insulin content and weak secretory responses to glucose in BRIN-BD11 cells [29,30]. Also, in contrast with the β-cells in the rat islets, the insulinoma INS-1E cells lack the characteristic intra-granular halo in their ISGs (shown by arrows in figure 3*c,d*), suggesting the dominance of an amorphous hormone inside these granules [3,21]. Such prevalence of an amorphous form of insulin in these permanent cells may result from an incomplete proinsulin → insulin conversion [31], as the proinsulin Zn^2+^-stabilized hexamers still remain soluble under granular pH of approximately 5.7, and only the cleavage of the C-peptide from proinsulin should yield low-solubility, crystallizable hexamers of the mature hormone [6,32,33].

Subsequently, the comparative ICP-OES and flow cytometry experiments revealed a reduction of zinc content in both permanent cell lines, in comparison with native rat islet cells (table 1, figure 6). This deficit can be ameliorated—to some extent—in the INS-1E cell line by higher medium Zn concentrations, but this drastically reduces the viability of these cells (figure 5). Moreover, supplementation of the medium with lower ‘safer’ (50 µM) levels of ZnCl_2_ does not restore the intracellular zinc levels in the permanent cell lines (figure 6*b,c*). Although the data of these observations are in general agreement with Nygaard *et al*. [28] and Chimienti *et al*. [34], who also used lower than 100 µM Zn^2+^ supplementation in INS-1E cells (in order to avoid zinc toxicity), our results indicate that even a much lower zinc concentration (approx. 20–50 µM) in the media is cytotoxic to these cells (figure 5). Moreover, Chimienti *et al*. [34] also did not observe any increase of intracellular zinc after supplementation of the media with 100 µM Zn^2+^. However, this small effect of 50–100 µM zinc supplementation is not surprising, if an endogenous, much higher Zn^2+^ concentration inside the ISGs (estimated as approximately 10–20 mM) is considered [5].

As the ISG-membrane-localized ZnT8 transporter is the most important zinc-transporting protein in the β-cells responsible for the translocation of the cytoplasmic Zn^2+^ into the ISGs [35,36], the expression of mRNA of this transporter was also measured in all our cell systems. The markedly decreased, impaired lower levels of ZnT8 mRNA in the INS-1E and BRIN-BD11 permanent cell lines (figure 10) suggest that ZnT8 deficiency could indeed contribute to low intracellular and intra-granular zinc levels.

It should be stressed, however, that the β-cells in the rat islets were not separated from other endocrine cell types (*α*, *δ*, *ε* and pancreatic polypeptide cells) also present in this tissue [37]. Although we attempted to separate these native β-cells from the collagenase-digested rat islets by fluorescent activated cell sorting (FACS) (data not shown), these experiments were unsuccessful owing to a prevalent destruction of all cells. Therefore, assuming that rat islets contain approximately 70–80% of the β-cells, [38], the actual difference in Zn^2+^ content between the rat islet β-cells and the permanent β-cell lines may probably be even higher. Such an assumption is further supported here by CFM (figure 7), which clearly indicated intensity differences in zinc-dependent fluorescence between permanent cells and pancreatic islets. Here, Zn^2+^-rich islets contain a distinctive, dense, green fluorescence that is concentrated in separate clusters in the β-cells (figure 7*f*).

As expected, immunoblotting experiments showed high levels of almost exclusively mature, processed insulin in the rat islets. By contrast, they indicated a much lower amount of insulin in INS-1E cells, and, on the threshold of detectability, in BRIN-BD11. What is interesting, besides insulin and proinsulin, INS-1E cells also contain other unidentified proteins with higher molecular weights (figure 8, proteins marked by asterisks). Despite repeated attempts of the N-terminal amino acid sequencing, tryptic digestion/MS analysis and immunoprecipitation, these proteins could not be unequivocally identified. However, the MS data of the proteolytic digests of the lower (10 kDa) band showed only the presence of insulin fragments and no other proteins (data not shown). This suggests that these higher molecular weight ‘aggregates’ could be oxidized, misfolded forms of insulin (or proinsulin), cross-linked by the mismatched disulfide bonds. The eminent presence of proinsulin in INS-1E cells indicates an aberrant (pro)hormone folding/processing there, which is also observed in insulinomas, and resulting there in the formation of both proinsulin- and insulin-containing secretory granules [39–41].

Finally, we also showed a great under-expression of insulin mRNA (figure 9), in both permanent cell lines; approximately 70-fold (in INS-1E) and approximately 30 000-fold (in BRIN-BD11) lower, than in rat islets (figure 9). This coincides with our immunodetection-assessed insulin levels in the corresponding cell samples (figure 8), also being in good agreement with our determination of the total insulin content; approximately 100-fold and approximately 35 000-fold lower than in rat islets in the INS-1E and BRIN-BD11 cells, respectively (figure 11). To assure a relevant comparison of the insulin content, we used a similar—10^2^ islets versus 10^5^ cells—amount of the material.

In summary, our data presented here clearly indicate that the reduced intracellular zinc levels in the permanent INS-1E and BRIN-BD11 cells are correlated with an impaired insulin production, and with a much lower expression of ZnT8 mRNA. However, the exact molecular nature of the mechanism by which zinc levels/ZnT8 deficiency can affect, or even cause, an altered insulin production and its processing in these permanent cell lines remains unclear. Nevertheless, it cannot be excluded that, considering the important role of Zn^2+^ in insulin oligomerization *in vitro,* its deficiency can impact the effective formation of proinsulin hexamers, therefore having a direct, causative effect on proinsulin processing.

This suggests a more extensive role of Zn^2+^ ions in insulin production, besides their oligomer-stabilization/storage role for insulin hexamers and their co-secretion. Moreover, Zn^2+^ ions are also involved in paracrine and autocrine communication within the pancreas [6], as, for example, the repression of glucagon-secreting activity of neighbouring α-cells [42], contributing also to the regulation of β-cell mass as a potent inhibitor of the apoptosis [43,44]. Significantly, it also seems that the disruption of zinc homeostasis is correlated with impaired insulin sensitivity and signalling, as is often observed in both type 1 and type 2 diabetes. For example, diabetic patients often display hypozincaemia [45] or hyperzincuria [46], while zinc-deficient rats also exhibit reduced insulin secretion and glucose sensitivity [47].

Therefore, our results presented here confirm and expand the ubiquitous role of Zn^2+^ ions in insulin molecular and cell biology, and underline the need for further in-depth studies of their inter-relationship. However, they also underline and amplify the methodological limitations of the rat permanent β-cells and their application to structural studies. This is of special importance for a reliable *in vivo* model of the β-cell for effective diabetes research and clinical applications.

## 4. Methods

### 4.1. Cell cultures

The rat clonal beta cell line INS-1E [48] AddexBio, San Diego, CA, USA; Cat no. C0018009), derived from parental INS-1 cells [49], was cultured in a humidified atmosphere containing 5% CO_2_ and at 37°C in a complete medium, composed of RPMI 1640 medium supplemented with 10% heat-inactivated fetal bovine serum (FBS), 1 mM sodium pyruvate, 50 mM 2-mercaptoethanol, 2 mM l-glutamine, 10 mM HEPES, 100 U ml^−1^ penicillin and 100 U ml^−1^ streptomycin.

The clonal beta cell line BRIN-BD11 (Sigma-Aldrich, St Louis, MO, USA; Cat no. 10033003) is a hybrid cell line, formed by the electrofusion of a primary culture of rat pancreatic islets (New England Deaconess Hospital, NEDH) with RINm5F cells [50,51]. The cell line was maintained in a humidified atmosphere containing 5% CO_2_ at 37°C in a complete medium, composed of RPMI 1640 medium supplemented with 10% heat-inactivated FBS, 2 mM l-glutamine, 100 U ml^−1^ penicillin and 100 U ml^−1^ streptomycin.

### 4.2. Isolation of rat pancreatic islets

Islets from male Wistar rats (approx. 400–500 g, 6 months old, Velaz s.r.o., Czech Republic) were isolated using a previously described method [52]. Six to eight animals were used for isolation islets only, and 40 animals were used for isolation of islets for isolation of ISGs (see below). Briefly, the main pancreatic duct in an anaesthetised animal was cannulated. After donor euthanizing, the pancreas was filled with 15 ml of collagenase (Sevapharma, Prague, Czech Republic, 1 mg ml^–1^ in HBSS buffer), before being excised and incubated for 30 min at 37°C. After removal of fat and floating tissue by centrifugation in HBSS (110*g*, 5 min, 4°C), the digest was passed through a sterile 450 µm sieve. Islets were purified using discontinuous Ficoll (Ficoll 400 DL, Sigma-Aldrich, USA) density gradient composed of four layers (1.108 g ml^−1^, 1.096 g ml^−1^, 1.068 mg l^−1^, 1.037 mg l^−1^) and centrifuged at 520*g* for 20 min. Isolated islets were then collected from the interlayer of discontinuous Ficoll gradient (1.068/1.096 mg l^−1^) and then washed twice in ice-cold Hank's balanced salt solution (HBSS) to remove Ficoll. Islets were kept overnight at 37°C in 5 ml of CMRL medium (Cellgro; Mediatech, Manassas, VA, USA) supplemented with 10% FBS or immediately used for experiments.

All animal experiments described in this study were performed according to the ethical guidelines for animal experiments and to the EU (86/609/EU) and Czech Republic Law 246/1992 and were approved by the Committee for experiments with laboratory animals of the Academy of Sciences of the Czech Republic (decision no. 38/2013 was issued on 8/4/2013).

### 4.3. Isolation of rat pancreatic islet cells

For disintegration of islets to single cells, cultured islets were rinsed in phosphate buffered saline (PBS) and incubated in Accutase (Sigma-Aldrich, USA) for 10 min with occasional pipetting at room temperature (RT). A pellet of cells (approx. 90% of single cells) was resuspended in HBSS buffer, centrifuged at 120*g* for 4 min. The excess of HBSS buffer was removed. The procedure was repeated twice. The resulting pellet of dispersed islet single cells was immediately used in experiments.

### 4.4. Isolation of secretory granules from INS-1E cells and pancreatic islets

The isolation of ISGs was performed according to the protocol published by Brunner *et al*. [16]. INS-1E cells were grown in 150 cm^2^ dishes until about 6 × 10^8^ cells were obtained, or islets from 40 rats were used. All the procedures were performed at 4°C. Cells were washed once with PBS and scraped in cold PBS. Cells were then homogenized in 15 ml of SMT buffer (0.27 M sucrose, 10 mM MOPS/1 M Tris, pH 6.8) by three strokes through a 21-G needle, followed by three strokes through a 25-G needle in order to break the cells. Cell debris and nuclei were removed by centrifugation for 5 min at 1000*g*. The supernatant was transferred to a new tube, and the remaining pellet was homogenized in 15 ml of SMT by three strokes through a 21-G needle, followed by five strokes through a 25-G needle. The homogenate was centrifuged for 5 min at 1000*g*, and the resulting supernatant was pooled with the first one. The pooled supernatants were finally centrifuged for 10 min at 1000*g* to obtain the post-nuclear supernatant (PNS). The PNS was centrifuged at 24 700*g* for 20 min to separate organelles from the cytosol. The resulting pellet was resuspended in SMT, loaded on a discontinuous Nycodenz gradient composed of three layers (23.4, 8.8 and 4.4% w/v Nycodenz in SMT buffer, pH 6.6) and centrifuged at 107 000*g* for 75 min. The presence of insulin granules within the gradient was followed, using dot-blot with anti-insulin antibody (L6B10, Cell Signalling). The cloudy ISG fraction (at about one-third of the tube height, fractions N8-N10 in figure 1) was recovered and the volume was adjusted to 10 ml with SMT. This suspension was loaded on 27% w/v Percoll solution, which was centrifuged at 35 000*g* for 45 min. The ISG fraction near the bottom of the tube (fraction P13 in figure 1) was recovered and washed three times with 10 ml of SMT and centrifuged at 30 000*g* for 20 min. The ISGs were finally re-suspended in 100 µl of SMT. Protein content was determined by the Bradford assay. The isolated granules were separated on SDS-PAGE (12% gels) and checked for insulin and betagranin (anti-chromogranin A antibody directed against N terminal amino acids, ab45179, abcam) content, using the standard Western blot procedure.

### 4.5. Chemical fixation of cell samples and transmission electron microscopy

INS-1E cells, BRIN-BD11 cells and freshly isolated rat islets were fixed with 2.5% glutaraldehyde in 0.1 M cacodylate buffer (pH 7.5) at RT for 15 min. Then the cells were harvested and centrifuged at 800*g* for 5 min and islets were centrifuged at 120*g* for 4 min. A new batch of 2.5% glutaraldehyde in 0.1 M cacodylate buffer was added and then the pellets were post-fixed in 1% osmium tetroxide, dehydrated in ethanol and embedded in Agar 100 epoxy resin. Ultrathin sections (70 nm) of cells were cut with a diamond knife on a Leica UC6 ultra microtome (Leica Microsystems, Wetzlar, Germany). The thin sections were collected on Parlodion-coated microscopy grids and contrasted, using saturated uranyl acetate and lead citrate [53]. The samples were analysed with a transmission electron microscope JEOL JEM-1011 device at 80 kV beam acceleration voltage.

### 4.6. Determination of zinc content in INS-1E cells by atomic absorption spectroscopy (inductively coupled plasma-optical emission spectroscopy)

Briefly, 10^6^ INS-1E cells were plated in six-well plates and the cells were treated with 0.0–1.0 mM ZnCl_2_ in complete media for 72 h. The duplicates of each concentration were used. After 72 h, the cells were trypsinised, counted and washed three times with PBS. Then 10^5^ cells from each well were diluted with PBS and the intracellular zinc concentration in cells was determined by ICP-OES. The ICP-OES measurements were performed using the SPECTRO ARCOS optical emission spectrometer (SPECTRO Analytical Instruments, Kleve, Germany) with a radial plasma observation. The SPECTRO ARCOS features a Paschen-Runge spectrometer mount; the wavelength range between 130 and 770 nm can be simultaneously analysed. Zinc ions were determined using analytical line at 213.85 nm.

### 4.7. Analysis of isolated insulin secretory granules from INS-1E cells and rat pancreatic islets by X-ray fluorescence

Isolated rat islets and ISGs from INS-1E cells were loaded into special X-ray capillaries (1.0 mm diameter, 0.01 mm wall thickness (Capillary Tube Supplies, Bodmin, UK)), and their wide ends were sealed with Parafilm. They were then mounted in custom-made foam-filled 50 ml plastic containers (Sarsted) and centrifuged for 8 h at 3800*g*. The foam filling and the *g* force/time was optimized to prevent capillary breaking, while allowing the concentration of the biological material. The supernatant was discarded and the capillaries were cut close to the precipitate of the granules and sealed with hot wax. They were mounted on the goniometer at Diamond Light Source (Didcot, UK) and two XRF spectra were taken [54]. The energy of the incoming X-ray beam was at 12.658 keV. The exposure time was fixed at 1 s, while the beam attenuation was set so as to avoid saturation of the fluorescence detector.

### 4.8. Flow cytometry

INS-1E and BRIN-BD11 cells were cultured for 72 h in 24-well plates in a complete RPMI medium, supplemented or not with 50 µM ZnCl_2_. The medium was removed, and cells were rinsed by fresh RPMI and subsequently incubated in trypsin/EDTA solution for 5 min to release adherent cells from the surface. Cells were transferred into HBSS buffer and centrifuged at 250*g* for 5 min. This procedure was repeated twice. In parallel, the fresh single-cell suspension from rat pancreatic islets was prepared as described above.

Thereafter, the cell samples were incubated (at 10^5^ cells per 100 µl) for 30 min on ice with 5 µM zinc-specific fluorescent probe FluoZin-3AM (Invitrogen) and 30 nM Pluoronic F-127 (Invitrogen) in the HBSS buffer (Thermo Fisher Scientific). In parallel, to determine the specificity of FluoZin3-AM for chelatable Zn^2+^, the cells were also incubated for 30 min at RT with 50 µM zinc chelator N,N,N’,N’-tetrakis-(2-pyridylmethyl) ethylenediamine (TPEN, Sigma-Aldrich, USA) followed by incubation with FluoZin-3AM and Pluoronic F-127. After incubation, the samples were washed twice with HBSS buffer again. Then, 100 µl of cell suspension (containing approximately 10^5^ cells) was placed into wells of a polypropylene 96-well plate (round bottom). Samples were analysed with a BD LSR Fortessa™ cell analyser (Becton, Dickinson and Company) operated by BD FACS Diva™ Software. The gates on the side scatter and forward scatter were set to ensure the measurement of viable cells, and 10 000 events were measured for each well. All variants of experiments (different staining agents, both cell lines and rat pancreatic islet cells) were performed in triplicates or tetraplicates and the results were presented as means with standard deviations.

### 4.9. Confocal microscopy of fluorescently labelled cells

INS-1E and BRIN-BD11 cells were grown in four-chamber 35 mm glass bottom dishes (In Vitro Scientific). After 3 days, the cells were washed twice with HBSS buffer and one chamber of each dish was treated with 50 µM TPEN for 30 min. Thereafter, the cells were incubated with 5 µM FluoZin-3AM (Invitrogen) and 30 nM Pluoronic F-127 in HBSS buffer for 30 min at RT. Finally, the cells were washed twice with 200 µl of HBSS.

Confocal images (pinhole 1 Airy unit) of cells in each chamber were taken with a LSM 780 confocal microscope (Carl Zeiss Microscopy), using an oil-immersion objective (Plan-Apochromat 63×/1.40 Oil DIC M27). The fluorescent images were collected at RT, using 4.5% of the 405 nm diode laser (max. power 30 mW) for excitation, with emission collected from 410 to 585 nm (voltage on detector 850 V) for Hoechst 34580, and 4.0% of the 488 nm argon-ion laser (max. power 25 mW) for excitation, with emission collected from 517 to 534 nm (voltage on detector: 870 V) for ATTO488. All images were taken using the same settings. The FluoZin-3AM maxima for excitation and emission are at 488 nm and 525 nm, respectively. The microscope was operated and the images were processed with ZEN 2011 software (Carl Zeiss Microscopy).

### 4.10. Tricine-sodium dodecyl sulphate-polyacrylamide gel electrophoresis

One hundred isolated islets (about 1 000 cells per islet) and 100 000 cells from both cell lines (INS-1E and BRIN-BD11) were dissolved in denaturing buffer (7 M urea, 2 M thiourea, 4% (w/v) CHAPS and 50 mM dithiothreitol, 0.8%). The protein content was determined using the Bradford assay. Cell lysates were analysed using electrophoresis. Separation of proteins smaller than 15 kDa was achieved, using 16% acrylamide separation gel in combination with 10% spacer gel and 4% stacking gel in a Tricine-Tris buffer system essentially according to Schägger *et al*. [55]. Proteins were transferred to 0.2 µm PVDF membrane (BioRad) in 25 mM Tris, 192 mM glycine and 20% (v/v) transfer buffer pH 8.3 at 12 V, 0.4 mA cm^−2^ overnight. Blots were developed by standard protocol, using anti-insulin antibody (L6B10, Cell Signalling) and anti-actin (20–33) antibody (Sigma).

### 4.11. RNA extraction and complementary DNA synthesis

Extraction and purification of RNA from cells or pancreatic islets was performed, using RNAzol RT (Sigma-Aldrich, USA) according to the manufacturer's instructions. RNA concentration was measured by ultraviolet absorbance at 260 nm with Nanodrop2000 (Thermo Fisher Scientific, USA). After extracting the total RNA, 500 ng of RNA was transferred to a new tube and heated up to 65°C for 5 min and then, transferred to ice immediately to denature the secondary structure of RNA and to improve reverse transcription efficiency. First-strand complementary DNA (cDNA) was synthesized from 500 ng of total RNA, using ReadyScript cDNA synthesis Mix (Sigma-Aldrich, USA) as described in the product manual. Briefly, ReadyScript cDNA Synthesis Mix was added to RNA samples and incubated at 25°C for 5 min, and next at 42°C for 30 min, at 85°C for 5 min, then held at 4°C. After completion of reverse transcription, the synthesized cDNA was five times diluted with water.

### 4.12. Quantitative real-time reverse transcription-polymerase chain reaction

Real-time reverse transcription-polymerase chain reaction (RT-PCR) was performed using the LightCycler480 (Roche Diagnostics, Mannheim, Germany). Final primer concentrations in the reaction mixture were 0.5 µM. The reversely transcribed reaction product (corresponding to 20 ng of RNA) in 2 µl was applied on LightCycler 8-tube strips (Roche Diagnostics, Germany). The total reaction volume was 20 µl. PCR was performed with the LightCycler 480 SYBR Green I Master (Roche Diagnostics) and by monitoring an increase in fluorescence of the SYBR Green Dye. cDNA was amplified by PCR with the following primers: glyceraldehyde-3-phosphate dehydrogenase (gapdh), direct 5′ – caccatcttccaggagcgag – 3′, reversed 5′ – ggcggagatgatgacccttt – 3′, ZnT8 direct 5′ – tcgagcagagatcctcggt – 3′, reversed 5′ – caagatgccgttggtgcaaa – 3′ and rat insulin 1 (Ins1) and 2 (Ins2), direct 5′ – accatcagcaagcaggtcat – 3′, reversed 5′ – gtttgacaaaagcctgggca – 3′. Rat insulins 1 and 2 share similar mRNA sequences, which allows one primer pair to measure the total insulin mRNA expression level. mRNAs of insulin and ZnT8 were normalized against GAPDH. The identity of the PCR products was confirmed by carrying out electrophoresis on 2.0% agarose gels.

### 4.13. Analysis of total insulin in BRIN-BD11 and INS-1E cells and islets of Langerhans

The total content of insulin inside the cells was measured by acid ethanol extraction [56]. The 10^2^ islets or the 10^5^ cells were sonicated in 30 µl of water for 15 s. The sonicates were mixed with acid ethanol (0.18 M HCl in 96% ethanol) in 1 : 3 proportion of sonicate and acid ethanol (90 µl). The mixed solutions in a total volume of 120 µl were incubated at 4°C for 12 h. After incubation, the samples were centrifuged at 14 000*g* for 5 min and the supernatants transferred to new tubes. The total insulin in the supernatants was measured by a sensitive rat insulin RIA (Millipore, Missouri, USA), according to the manufacturers' instructions.

## Data accessibility

The paper contains data from standardized methods. Blots (Dot-blot, Western blot) and tricine-SDS-page, data from transmission electron microscopy, XRF, atomic absorption spectroscopy, flow cytometry, confocal fluorescence microscopy and RNA expression. The data were evaluated using GraphPad Prism 8.
